# Three-dimensional morphogenesis of MDCK cells induced by cellular contractile forces on a viscous substrate

**DOI:** 10.1038/srep14208

**Published:** 2015-09-16

**Authors:** Misako Imai, Kazuya Furusawa, Takeomi Mizutani, Kazushige Kawabata, Hisashi Haga

**Affiliations:** 1 Transdisciplinary Life Science Course, Faculty of Advanced Life Science, Hokkaido University, N10-W8, Kita-ku, Sapporo 060-0810, Japan; 2 Research Center for Cooperative Projects, Hokkaido University Graduate School of Medicine, Sapporo, 060-8638, Japan

## Abstract

Substrate physical properties are essential for many physiological events such as embryonic development and 3D tissue formation. Physical properties of the extracellular matrix such as viscoelasticity and geometrical constraints are understood as factors that affect cell behaviour. In this study, we focused on the relationship between epithelial cell 3D morphogenesis and the substrate viscosity. We observed that Madin-Darby Canine Kidney (MDCK) cells formed 3D structures on a viscous substrate (Matrigel). The structures appear as a tulip hat. We then changed the substrate viscosity by genipin (GP) treatment. GP is a cross-linker of amino groups. Cells cultured on GP-treated-matrigel changed their 3D morphology in a substrate viscosity-dependent manner. Furthermore, to elucidate the spatial distribution of the cellular contractile force, localization of mono-phosphorylated and di-phosphorylated myosin regulatory light chain (P-MRLCs) was visualized by immunofluorescence. P-MRLCs localized along the periphery of epithelial sheets. Treatment with Y-27632, a Rho-kinase inhibitor, blocked the P-MRLCs localization at the edge of epithelial sheets and halted 3D morphogenesis. Our results indicate that the substrate viscosity, the substrate deformation, and the cellular contractile forces induced by P-MRLCs play crucial roles in 3D morphogenesis.

3D morphogenesis is an essential process for various *in vivo* phenomena such as embryonic development and tissue formation. Epithelial cells composing tissues receive mechanical stimuli from the extracellular matrix (ECM). Mechanical properties of the ECM such as stiffness, flexibility, and geometrical constraints are understood as factors that affect the cell behavior^1^,^2^,^3^,^4^,^5^,^6^,^7^,^8^. For instance, when focusing on substrate stiffness, MDCK cells migrate collectively toward one direction when cultured on a soft collagen gel^3^. It is also reported that the elasticity of the basement membrane is necessary to form the structures that accompany tissue compartmentalization in the zebrafish development^9^. A recent study reveals that epithelial cell directional motility is improved when the contribution of the substrate viscosity is larger than that of its elasticity^6^. Furthermore, in computer simulations, 3D morphological change is induced by the substrate viscosity around cell masses^10^. Nevertheless, the relationship between living cell 3D morphogenesis and the substrate viscosity has not been clarified.

Multicellular morphology is regulated by cellular contractile forces of stress fibres, which consist of actin and myosin II filaments^11^,^12^,^13^,^14^,^15^,^16^,^17^,^18^. The phosphorylation of myosin II regulatory light chain (MRLC) generates the cellular forces. There are two phosphorylation sites in MRLC, threonine 18 and serine 19. Serine 19 is phosphorylated prior to threonine 18^19^. It is well-known that di-phosphorylation of MRLC enhances the degree of elasticity and the traction force of a single cell^20^. The signalling pathways involved in MRLC phosphorylation have been previously described. For instance, the small G protein, RhoA, activates the Rho-associated protein kinase (Rho-kinase), which then phosphorylates MRLC^21^. Alternatively, myosin II light chain kinase (MLCK) is also known as an MRLC kinase, which is regulated by calcium and calmodulin^22^,^23^.

The relationship between morphogenesis and cellular contractile forces was recently investigated *in vivo* and *in vitro*. During tissue invagination and furrow formation during the development process, cellular contractile forces support the proper morphogenesis. In the development of a mouse lens, the balanced activities of small G proteins, Rac1 and RhoA, were shown to control cell shape and support proper lens morphogenesis^24^. Moreover, the inhibition of RhoA, ROCK, and myosin II activities in MDCK cells led to the inversion of the orientation of epithelial cell polarity, resulting in abnormal cyst formation^12^. Despite the fact that epithelial cells are well known to present various morphologies, the mechanism underlying morphogenesis remains unclear.

In this study, we cultured MDCK epithelial cells on a viscous substrate. The cells presented a tulip hat-like 3D morphology induced by the deformation of the peripheral substrate via cellular contractile forces. We revealed that the 3D tulip hat-like morphology changed in a substrate viscosity-dependent manner. In addition, the cellular contractile forces generated in the edge of the cell sheets were required for the tulip hat-like morphogenesis.

## Results

### Matrigel substrate viscosity

To modify the matrigel substrate viscosity, we used genipin (GP), a cross-linker of amino group^25^,^26^,^27^,^28^. We assumed that increasing cross-links by GP would increase the matrigel viscosity. The GP solution was mixed with the matrigel before solidification at the following concentrations, 0, 0.25, and 0.50 mM (referred to as non-treated (NT)-Matrigel, 0.25-GP-Matrigel, and 0.50-GP-Matrigel, respectively).

**Figure 1:**
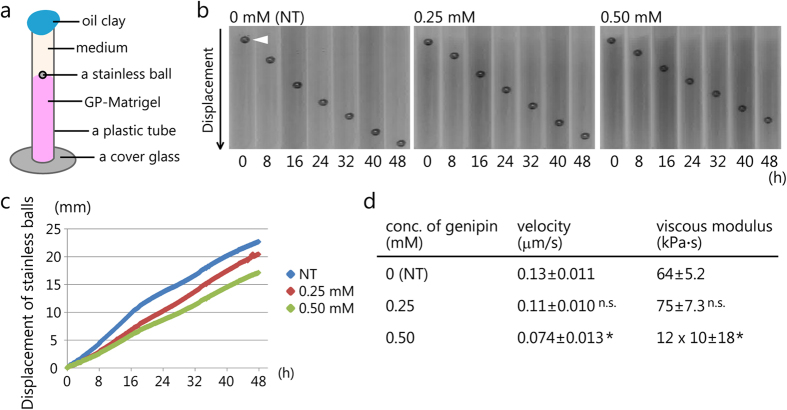
Measurement of the matrigel viscosity after genipin treatment. (**a**) Schematic representation of matrigel viscosity measurement conditions. The matrigel used for the measurement was poured into a cylinder built from a plastic tube and a cover glass prior to solidification. After solidification, the gel surface was filled with medium. The radius of the cylinder is 1.5 mm, and its length is approximately 50 mm. The radius of the stainless ball is 0.75 × 10^−3^ m. The cylinders were sealed with oil clay to prevent changes in the medium pH. (**b**) Kymographs of the stainless balls in the matrigel. The genipin concentrations in the matrigel are indicated above each image: 0 mM (No-treatment; NT), 0.25 mM, and 0.50 mM. Numbers under the images represent the time relative to the start (0 h) and the end (48 h) of the observation. White arrowhead shows the initial position of the stainless ball. (**c**) Time-displacement plot of stainless balls through the matrigel. The displacement in the plot corresponds to the result obtained from the time-lapse observations in Fig. 1b. The horizontal axis is the relative time and the vertical axis is the relative displacement of stainless balls. (**d**) Mean velocities of stainless balls and the matrigel viscous moduli. The mean velocities are the gradients of the linear approximate equations. This experiment was repeated three times. Each value represents the mean value ± standard error of the mean (s.e.m.). Statistical significance between NT and other two samples: n.s., p ≥ 0.05, *p < 0.05.

To measure the viscosity of the matrigel substrata, we performed an experiment in which a stainless ball was dropped into the matrigel. This experiment is referred to as the ball dropping method hereafter. To observe the stainless ball in the matrigel, we built a thin cylinder by assembling a plastic tube and a cover glass (Fig. 1a). The radius of the cylinder is 1.5 mm, and its length is approximately 50 mm. GP-Matrigel substrata in the cylinders were incubated at 37 °C overnight to solidify and for the cross-linking to occur. After incubation, a stainless ball was set on the gel upper surface. The cylinders were subsequently filled with medium and sealed with oil clay (Fig. 1a). The sample cylinders were kept in a styrofoam box to maintain the temperature at 37 °C. We obtained time-lapse images captured with a camera every 10 min for 48 h (Fig. 1b and Supplementary Movie S1). We estimated fall velocities (*v*) from a linear approximate equation of the displacement-time graph (Fig. 1c) and calculated viscous moduli (η) using the following equation:


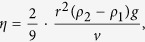


where *r* is radius of the stainless ball, ρ_1_ and ρ_2_ represent the density of the matrigel and the stainless ball, respectively, and *g* is the acceleration of gravity, and their values are as follows: r = 0.75 × 10^−3^ m, ρ_1_ = 1.1 × 10^3^ kg/m^3^, ρ_2_ = 0.79 × 10^3^ kg/m^3^, and g = 9.8 m/s^2^. The viscous moduli and estimated fall velocities are described in Fig. 1d. The viscous moduli of 0.25-GP-Matrigel and 0.50-GP-Matrigel were 1.18–fold and 1.86–fold higher than that of NT-Matrigel, respectively. These results suggest that the matrigel viscosity increased with increasing concentrations of GP.

### 3D morphology of MDCK cells on the matrigel substrate

**Figure 2:**
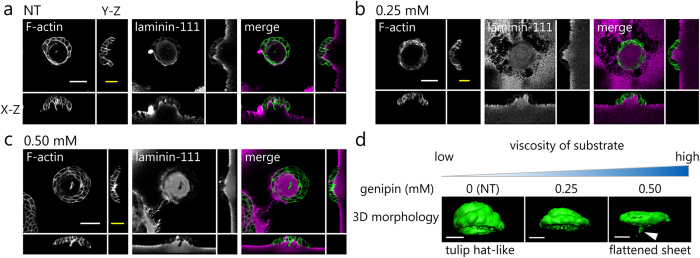
MDCK cell morphological change on genipin-treated matrigel. (**a**–**c**) Immunofluorescence images of MDCK cells on genipin-treated matrigel. The concentrations of genipin are (**a**) 0 mM (NT), (**b**) 0.25 mM, and (**c**) 0.5 mM. The cells and matrigel surface structure are detected by F-actin and laminin-111 staining, respectively. Cross-sectional views are shown together. Scale bar (white) = 50 μm. Scale bar (yellow) = 20 μm. (**d**) Relationship between cell morphology and substrate viscosity. The images of 3D morphology were reconstructed using the results presented in (**a**–**c**). White arrowhead shows the protrusion-like structure in the 3D structure. Scale bar = 30μm.

To observe MDCK cell morphologies on the NT-Matrigel substrate, we cultured the cells for 4 days and performed immunofluorescence staining for F-actin and laminin-111. Laminin-111 is a major component of the matrigel. Thus, we can identify the substrate surface. The F-actin staining indicated that MDCK cells formed a 3D structure resembling a tulip hat on the NT-Matrigel (Fig. 2a,d). Moreover, laminin-111 staining showed that the inside of the tulip hat was filled with the matrigel substrate (Fig. 2a). This result indicates that the MDCK cells grown on the NT-Matrigel deformed the substrate and formed a 3D tulip hat-like morphology.

### MDCK cell 3D morphological changes depend on the matrigel viscosity

To investigate the relationship between cell morphology and substrate viscosity, MDCK cells were cultured on the different GP-Matrigels for 4 days. F-actin and laminin-111 staining indicated that MDCK cells on 0.25-GP-Matrigel formed a 3D structure similar to that of the cells cultured on NT-Matrigel. However, the height of the structure above the matrigel surface was lower compared with that the structure obtained on NT-Matrigel (Fig. 2d). In 0.50-GP-Matrigel, MDCK cells did not present a 3D tulip hat-like morphology, but formed a monolayer sheet (Fig. 2c,d). Additionally, a protrusion was found beneath the monolayer sheet (Fig. 2d, white arrowhead). While we previously reported the existence of a protrusion with cells cultured on matrigel within 2 days^29^, the reason why the protrusion appeared remains unclear. The relationship between cell morphology and the substrate viscosity is shown in Fig. 2d. These results imply that MDCK cell morphology varied according to the substrate viscosity.

### Substrate deformation is induced by cellular contractile forces

**Figure 3:**
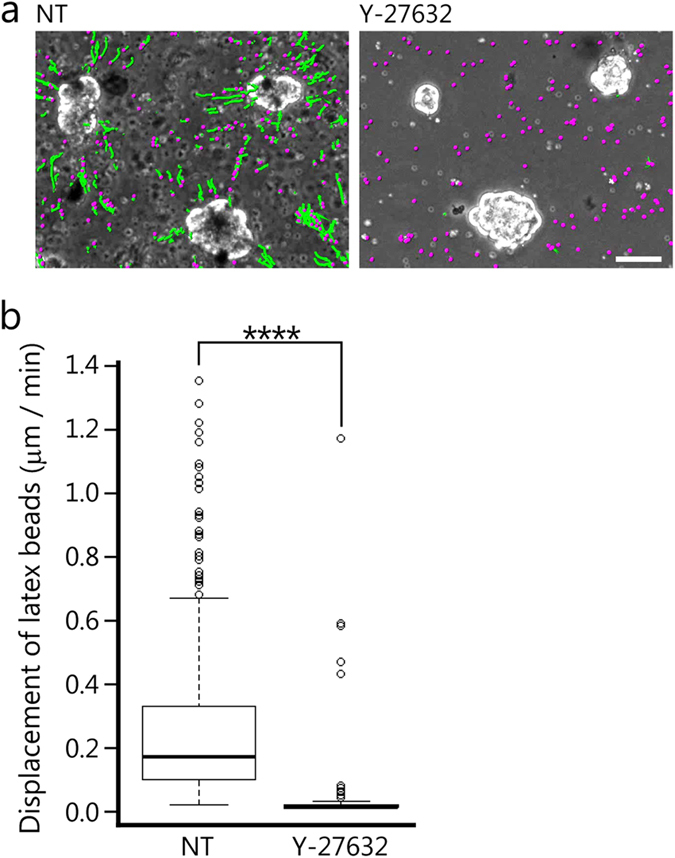
The matrigel surface is deformed during the 3D morphogenesis. (**a**) Trajectories of latex beads embedded in the matrigel. The beads are shown as magenta spheres and the trajectories of the beads are shown as green lines. The green lines represent the displacement for 1.5 h. NT: No reagent control, Y-27632: ROCK inhibitor (10 μM). For this analysis, more than 50 latex beads were randomly chosen from each condition. Scale bar = 50 μm. (**b**) The box plot shows the displacement of embedded latex beads. Error bars represent s.e.m. ****p < 0.0001.

The staining of the matrigel surface revealed that the pericellular substrate was deformed. To observe the deformation of the matrigel surface during 3D morphogenesis, we embedded latex beads in the matrigel and followed the bead movement and the matrigel deformation. Time-lapse observations for 24 h showed that the latex beads moved toward the cell masses. Green lines in Fig. 3a and Supplementary Movie S2 exhibit the beads trajectories that occurred within 1.5 h. This result indicates that MDCK cells exerted forces on the matrigel and deformed the peripheral substrate. Thus, we postulated that the matrigel deformation resulted from the cellular contractile forces. We then blocked the cellular contractile forces by treatment with inhibitors and conducted time-lapse observations. We treated the cells with a Rho-kinase inhibitor (Y-27632; 10 μM). Y-27632 promotes the dephosphorylation of MRLC^30^. Time-lapse observation showed that the displacement of latex beads was halted (Fig. 3a and Supplementary Movie S3). The median value of displacement velocity was about 10 times slower than that in NT conditions (Fig. 3b, ****p < 0.0001). These results suggest that the cellular contractile forces induced by MRLC phosphorylation are involved in the matrigel deformation.

### The cellular contractile forces are involved in 3D morphogenesis on matrigel

**Figure 4:**
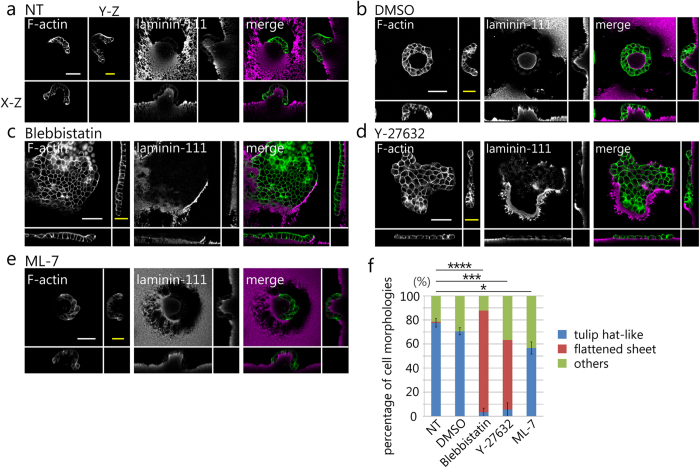
Morphological change of MDCK cells treated with inhibitors. (**a**–**e**) Immunofluorescence staining of the cells and matrigel surface. The conditions are the following: (**a**) No-treatment (NT), (**b**) DMSO as a control for the following reagents except for Y-27632, (**c**) Blebbistatin, (**d**) Y-27632, and (**e**) ML-7. Orthogonal views are shown together. Scale bar (white) = 50 μm. Scale bar (yellow) = 20 μm. Three colonies were randomly chosen in each experiment (N = 3 (at least)). (**f**) The graph indicates the proportion of cell structures. Cell structures are categorized into three morphology types: tulip hat-like structure, monolayer sheet, and others. Thirty colonies were randomly chosen in three independent experiments (N = 3). Results are shown as mean value ± s.e.m. *p < 0.05, ***p < 0.001, ****p < 0.0001.

To examine whether cell morphology varies by blocking the cellular contractile forces, we observed cell morphology on the matrigel in the presence of inhibitors. The cells were treated with inhibitors of myosin II activity (Blebbistatin; 10 μM), Rho-kinase (Y-27632; 10 μM), and MLCK (ML-7; 2 μM) continuously for 3 days. The substrate deformation was blocked by treatment with blebbistatin and Y-27632, but not by ML-7 treatment (Fig. 4c–e). Furthermore, to compare the difference in cell morphology among the samples, we chose 30 colonies at random from one sample and categorized cell morphologies into three types, a tulip hat-like structure, a flattened sheet, and others. This analysis was repeated three times. The percentage of each structure is represented in Fig. 4f. Tulip hat-like structures accounted for 70–80% of the total cell morphology under NT and dimethyl sulfoxide (DMSO) conditions. In contrast, treatment with blebbistatin and Y-27632 caused a dramatic reduction of the number of tulip hat-like structures (****p < 0.0001, and ***p < 0.001) and increased the number of monolayer sheet structures. In ML-7 treatment conditions, the tulip hat-like structures accounted for approximately 60% of the total cell morphology, although there was a significant reduction in the number of tulip hat-like structure in comparison to NT conditions (*p < 0.05). Additionally, flattened sheet structures were not observed at all. These results imply that myosin II activation and MRLC phosphorylation by Rho-kinase are involved in the morphological changes observed on matrigel.

### Spatial distribution of phosphorylated MRLCs in the 3D tulip hat-like structure

**Figure 5:**
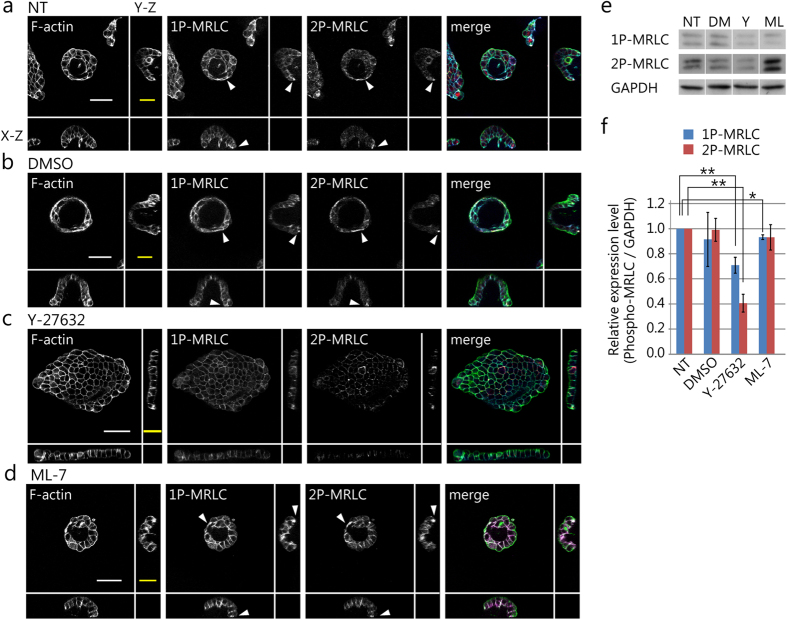
Localization and expression of phosphorylated-MRLC in the cells. (**a**–**d**) F-actin, 1P-MRLC, and 2P-MRLC staining on the matrigel surface. Each sample was treated with (**a**) no reagent (NT), (**b**) DMSO as a control, (**c**) Y-27632, and (**d**) ML-7. Cross-sectional views, X-Z, and Y-Z plane are shown together. White arrowheads in the images represent 1P-MRLC and 2P-MRLC localization. Three colonies were randomly chosen in each experiment (N = 3 (at least)). Scale bar (white) = 50 μm and scale bar (yellow) = 20μm. (**e**,**f**) Immunoblot and statistical analysis of 1P-MRLC and 2P-MRLC protein expression in MDCK cells cultured on matrigel-coated dishes. The cells cultured on non-treated matrigel were used as control. Mean values are calculated from three independent experiments. Error bars represent s.e.m. *p < 0.05, **p < 0.01.

To determine the area where the cellular contractile forces were exerted within the 3D structure, the cells were immunostained for phosphorylated MRLC (P-MRLC). P-MRLC localization represents the spatial distribution of the cellular contractile force. Immunofluorescence imaging was performed to observe P-MRLC in MDCK cell 3D morphology. MRLC has two phosphorylation sites, threonine 18 and serine 19. MRLC di-phosphorylation enhances the degree of elasticity and the traction force^4^,^20^. In NT and DMSO treated samples, we found that mono- and di-phosphorylated MRLC (1P-MRLC and 2P-MRLC) localized along the periphery of the tulip hat-like structure (Fig. 5a,b, arrowheads). 1P-MRLC and 2P-MRLC localization toward the outer edge of the epithelial sheet structure disappeared when the cells were treated with 10 μM Y-27632 (Fig. 5c). In addition, MDCK cells formed a flattened sheet. On the other hand, the cells formed a 3D tulip hat-like structure and the localization of both 1P- and 2P-MRLC was preserved when the cells were treated with ML-7 (Fig. 5d, arrowheads). Furthermore, we detected 1P-MRLC and 2P-MRLC phosphorylation in cells treated with Y-27632 and ML-7 by western blotting. Both 1P-MRLC and 2P-MRLC significantly decreased after treatment with 10 μM Y-27632 (**p < 0.01), and 1P-MRLC decreased after treatment with 2 μM ML-7 (*p < 0.05, Fig. 5e,f). These results indicate that 2P-MRLC localization toward the outer edge of the epithelial sheet is essential for the 3D tulip hat-like morphogenesis, though MRLC mono-phosphorylation also affects it.

## Discussion

**Figure 6:**
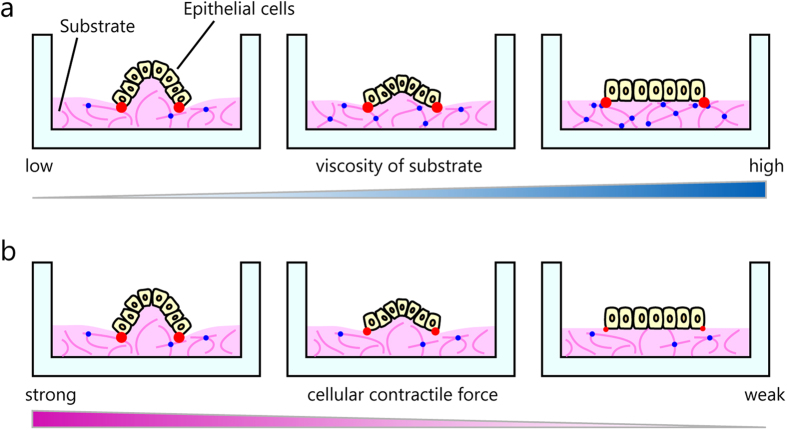
A proposed model for epithelial cell 3D morphogenesis induced by the substrate viscosity and cellular contractile forces. (**a**) The morphological change involved the substrate viscosity. The substrate viscosity was altered by the extent of cross-links between peptide chains contained in the matrigel. Pink lines represent the peptide chains. The blue dots represent the cross-linking sites in the peptide chains. Red dots indicate the localization of the cellular contractile force and their sizes indicate the strength of the cellular contractile force (**b**) 3D morphology on the viscous substrate changes depending on the strength of the cellular contractile force.

We suggest a model for epithelial cell 3D morphogenesis on a viscous substrate induced by the cellular contractile forces (Fig. 6). On viscous substrate like matrigel, epithelial cells form a 3D tulip hat-like structure. Moreover, as the substrate viscosity increased, the structure formed by epithelial cells changed to a flattened sheet structure (Fig. 6a). We also confirmed that deformation of the peripheral substrate decreased with increase in substrate viscosity (Supplementary Figure 1 and Supplementary Movie S4–6). Concomitantly, the cells exerted contractile forces at the edges of the cell sheets (Supplementary Figure 2 and Fig. 6a, indicated by red dots). The amount of cross-link formation (Fig. 6a, blue dots) contributes to substrate viscosity (Fig. 6a). Figure 6b shows the relationship between the multicellular morphology and the cellular contractile forces. The epithelial cells generate contractile forces at the edge of the cell sheet toward the centre that causes the matrigel deformation, resulting in epithelial cells forming 3D tulip hat-like structures (Fig. 6b). Collectively, our results suggest that the observed 3D morphogenesis involved three important factors, the substrate viscosity, the substrate deformation, and the cellular contractile forces induced by P-MRLC.

Epithelial cells grown on matrigel exerted cellular contractile forces on the peripheral substrate and changed the substrate shape (Figs 2a and 3a). On the other hand, it is known that epithelial cells on a collagen gel do not form 3D tulip hat-like structures and do not remodel the peripheral substrate^3^. To explain the cell-induced matrigel deformation, we focused on the difference in the protein cross-links formed in these gels. Biomimetic substrates such as matrigel and collagen type I gel are known as physical gels. The molecule bindings in the physical gel are unstable and are repeatedly dissociated and associated by external forces and thermal energy^28^,^31^,^32^. The gel physical properties depend on the formation of cross-links between proteins. Laminin and type IV collagen in the matrigel form mesh structures, whereas type I collagen forms fibrous structures^33^,^34^,^35^,^36^. The flexibility and fluidity of the substrate are controlled by the amount of cross-linking formation^6^,^37^. In the present study, the matrigel did not deform when the matrigel viscosity was increased by GP treatment (Fig. 2). The GP-induced a cross-link, which is a chemical binding that is robust and durable^26^,^27^,^28^,^38^. This result means that the substrate deformation was blocked because of a decrease in the fluidity induced by GP. Thus, we conclude that the cross-link structure largely affects the matrigel deformability. Consequently, the deformation of the pericellular matrigel by the cellular contractile forces is easier than that of the type I collagen gel.

Differences between collagen gel and matrigel are not limited to those of cross-linking formation. In the present study, we use matrigel, a biomimetic material, containing various proteins such as laminin-111, type IV collagen, and soluble growth factors. Collagen gel is a material composed of type I collagen only. Proteins and growth factors in the gel affect cell behaviour such as migration, morphology, and proliferation^39^,^40^,^41^. Therefore, in the future, we plan to design a viscous substrate that circumvents these issues.

The epithelial cells on the matrigel presented a 3D tulip hat-like morphology (Fig. 2a). *In vivo*, similar 3D structures are observed during notochord tubulogenesis, brain morphogenesis, and optic cup formation^15^,^42^,^43^. The epithelial cell sheets hollow and form convex structures, caused by basal constriction. Networks of actomyosin and microtubule, laminin secretion, and focal adhesion components are the key factors of basal constriction^15^,^42^,^43^. These studies showed the importance of cell-substrate adhesion for basal constriction. Integrins localize at the plasma membrane and transduce signals from laminin to actomyosin via focal adhesion. We performed immunostaining for gp135, an apical marker, and laminin-β1, a basal marker. The results showed that the apico-basal polarity in the tulip hat-like structure was maintained (Supplementary Figure S3). Furthermore, we observed the localization of integrin β1 toward the basal surface of the tulip hat-like structure (Supplementary Figure S3). Hence, these results imply that the morphogenesis on matrigel can be induced by basal constriction.

In this study, 2P-MRLC localization toward the outer edge of the epithelial sheets was crucial for the 3D morphogenesis of a tulip hat-like structure. To elucidate whether there is a correlation between 2P-MRLC localization and 3D morphology, flattened epithelial sheets were cultured on matrigel and treated with Y-27632 as shown in Fig. 4d. After washing out Y-27632, the cell sheets were cultured for another 48 h. F-actin and 2P-MRLC immunostaining was performed to observe the 3D morphology and the cell force spatial distribution. The cells exerted contractile forces toward the edge of epithelial sheets after removing the inhibitor. We observed the sparse localization of 2P-MRLC throughout the epithelial sheets (Supplementary Figure S4). In addition, the cell sheets formed structures, which were similar to villi of the small intestine and the sulcus in the brain (Supplementary Figure S4). These results indicate that the 3D morphology depends on the substrate area on which the cells apply forces. We assume that the villus-like structure was formed due to the deformation generated by the sparse distribution of the cellular contractile force.

Recently, cell sheet transplant methods have been established in regenerative medicine^44^,^45^,^46^. Artificial organ formation requires that the cell sheets maintain their structures. To evaluate whether the tulip hat-like morphology is a persistent structure, we cultured MDCK cells on NT-Matrigel for 2 weeks and performed immunofluorescence staining of F-actin and laminin-β1, a basal marker. The results showed that the tulip hat-like morphology and cell polarity were maintained (Supplementary Figure 5). The epithelial cell sheet culture on viscous substrate will be applied to artificial organ formation in the future.

In conclusion, this study showed that 3D morphogenesis is induced by the substrate viscosity and the cellular contractile forces. We increased the matrigel viscosity by adding a cross-linking reagent. The 3D morphology on the matrigel changed due to the increment of the substrate viscosity. MDCK epithelial cells formed 3D tulip hat-like structures. Additionally, P-MRLC localization is important for 3D morphogenesis on viscous deformable substrates. Our data indicate that the substrate viscosity and the cellular contractile force are involved in morphological changes observed during 3D morphogenesis and believe that these factors will affect 3D morphogenesis *in vivo*.

## Methods

### Preparation of viscous substrata

We used matrigel as the extracellular matrix. Matrigel was extracted from Engelbreth-Holm-Swarm (EHS) mouse sarcoma, a tumour rich in extracellular matrix proteins, and stored at –20 °C. The matrigel was thawed overnight at 4 °C before use. In this study, we prepared three matrigel substrates, non-treated matrigel (NT-Matrigel), matrigel coat (CO-Matrigel), and genipin (Wako Pure Chemical Industries, Ltd., Osaka, Japan) -treated matrigel (GP-Matrigel). In NT-Matrigel, liquefied matrigel was poured into a sample dish and incubated for 30 min at 37 °C for solidification. In CO-Matrigel, the sample dish was covered with 10× diluted matrigel and incubated at 4 °C overnight. The method used for GP-Matrigel is described in the Results section.

### Cell culture

MDCK cells were obtained from the Riken Cell Bank and were cultured in Dulbecco’s Modified Eagle’s Medium (DMEM; Sigma, St. Louis, MO, USA) containing 10% bovine foetal serum (FBS; Equitech Bio Inc., Kerrville, TX, USA), and 1% antibiotic (Invitrogen, Carlsbad, CA, USA). Cells were incubated at 37 °C in a humidified incubator with 5% CO_2_. The trypsinized cell suspension was plated onto the substrate surface. After incubating for 4 days, the dishes were used for biological experiments.

### Inhibitors

We used Blebbistatin (Enzo Life Sciences, Farmingdale, NY, USA) to inhibit non-muscle myosin II, Y-27632 (Sigma, St. Louis, MO, USA) to inhibit Rho-kinase activity, and ML-7 (Sigma) to inhibit MLCK. Inhibitors were diluted in DMSO (Wako Pure Chemical Industries, Ltd.) except for Y-27632. We prepared a DMSO-treated sample as a control. The cells were treated with the following concentrations for 24 h or 4 days, 10 μM Blebbistatin, 10 μM Y-27632, and 2 μM ML-7.

### Time-lapse observations

The sample dishes were filled with culture medium and sealed with silicone grease to avoid changes in the medium pH. Time-lapse observations were conducted with a phase-contrast microscope (TE2000; Nikon, Tokyo, Japan) equipped with a 10× objective, which was kept in an acrylic resin box to maintain the temperature at 37 °C. Time-lapse images were captured every 5 min with Image-pro software (Media cybernetics Inc., Silver Spring, MD, USA). In matrigel deformation analysis, latex beads (2 μm; Polysciences, Inc., Warrington, PA, USA) were embedded in the matrigel to observe any deformations induced by the cellular contractile forces.

### Immunofluorescence

Cells were fixed with 1% or 2% formaldehyde in phosphate-buffered saline (PBS) for 5 min, permeabilized with 0.5% Triton-X100 in PBS for 5 min, and blocked with 0.5% bovine serum albumin (Sigma) or 0.5% skim milk (Megmilk Snow Brand Co., Ltd., Sapporo, Japan) in PBS. Reaction with the primary antibody was performed at room temperature overnight. The primary antibodies used were L9393 (Sigma) for laminin-111, anti-phospho-MRLC (Ser 19) mouse IgG (Cell Signaling Technology Japan, K.K., Japan), and anti-phospho-MRLC (Thr18/Ser19) rabbit IgG (Cell Signaling Technology) at a dilution of 1:250, 1:200, and 1:200, respectively. All samples were incubated with a mixed solution consisting of the secondary antibody and Alexa Fluor-488 phalloidin (Invitrogen) for F-actin staining at 37 °C for 1 h. AlexaFluor-594 goat anti-rabbit IgG (H + L) and AlexaFluor-546 goat anti-mouse IgG (H + L) were used as secondary antibodies (Molecular Probes, Eugene, OR, USA) at a concentration of 10 μg/mL. Fluorescent images were captured using confocal laser scanning microscopy (C1 confocal imaging system; Nikon). We used the Imaris software (Bitplane AG, Zürich, Switzerland) for 3D reconstruction of confocal images. This software enabled the visualization of 3D morphologies of the epithelial cells.

### Western blotting

We extracted proteins from the cells plated on matrigel-coated dishes. For fixation, the culture medium was removed from the dish and ice-cold 10% trichloroacetic acid (Sigma) in PBS was added. After washing with PBS, 200 μL SDS sample buffer (0.25 M Tris–HCl pH 8.8, 5% dithiothreitol, 2.3% sodium dodecyl sulphate, 10% glycerol, and 0.01% bromophenol blue) was added and the cells were lysed. Lysed cells were sonicated and heated at 95 °C for 5 min, and stored at−20 °C. We used 12.5% polyacrylamide gels for SDS-PAGE for 75 min and the proteins were transferred onto a polyvinylidene difluoride membrane (Millipore, Bedford, MA, USA). The membrane was blocked with 5% skim milk in TBST solution (10 mM Tris–HCl containing 150 mM NaCl and 0.05% Tween 20, pH 7.5). The primary antibodies used were anti-phospho-MRLC (Ser 19 or Thr18/Ser19; Cell Signaling Technology) rabbit IgG at 4 °C overnight. After three washes with TBST, HRP-conjugated anti-rabbit IgG antibody to reveal phosphorylated-MRLC and HRP-conjugated anti-mouse IgG antibody to reveal GAPDH were used as secondary antibodies at room temperature for 1 h. All antibodies were diluted in Can Get Signal Immunoreaction Enhancer (Solutions 1 and 2; Toyobo, Osaka, Japan). The target protein signal intensity was detected by using Immobilon Western Chemiluminescent HRP substrate (Millipore). GAPDH was used as an internal control, and its expression levels were measured. Image J software (National Institutes of Health, Bethesda, MD, USA) was used to quantify the signal intensity (Fig. 5d).

### Statistical analysis

Each experiment was repeated at least three times. The error bars represent the mean ± s.e.m. For viscoelasticity measurements and western blotting analysis, open source Image J (National Institutes of Health) with some plugins was used (Figs 1b,d and 5d). Displacement of latex beads embedded in the matrigel was analyzed by using Imaris software (Bitplane AG) (Fig. 3a and Supplementary Figure 2a), and was described by box-plot using R software (R Development Core Team, Vienna, Austria) (Fig. 3b and Supplementary Figure 2b). We performed all statistical analyses using student’s *t*-test, and considered a P-value of <0.05 as significant.

## Additional Information

**How to cite this article**: Imai, M. *et al.* Three-dimensional morphogenesis of MDCK cells induced by cellular contractile forces on a viscous substrate. *Sci. Rep.* **5**, 14208; doi: 10.1038/srep14208 (2015).

## Supplementary Material

Supplementary Movie S1

Supplementary Movie S2

Supplementary Movie S3

Supplementary Movie S4

Supplementary Movie S5

Supplementary Movie S6

Supplementary Information
