# Supplementary material for: Three-dimensional morphogenesis of MDCK cells induced by cellular contractile forces on a viscous substrate
Source: Sci Rep. 2015 Sep 16;5:14208. doi: 10.1038/srep14208 (PMC4571640; doi:10.1038/srep14208)
Supplement: Supplementary Information [file srep14208-s7.doc]

Supplementary Information

Title: Three-dimensional morphogenesis of MDCK cells induced by cellular contractile forces on a viscous substrate

Misako Imai, Kazuya Furusawa, Takeomi Mizutani, Kazushige Kawabata, and Hisashi Haga

Supplementary materials and methods

**Immunofluorescence antibodies**

The anti-laminin-β1 mouse IgG (2E8), anti-integrin-β1 (AIIB2) from Developmental Studies Hybridoma Bank (University of Iowa, Iowa City, IA, USA), and anti-gp135 mouse IgG (3F2; kindly provided by Prof. Ojakian) were used as primary antibodies at a dilution of 1:200, 1:200, and 1:300, respectively. Reaction with the primary antibody was performed at room temperature overnight. The secondary antibody and Alexa Fluor-488 phalloidin for F-actin staining were used as described in the Materials and Methods section.

Supplemental Figures


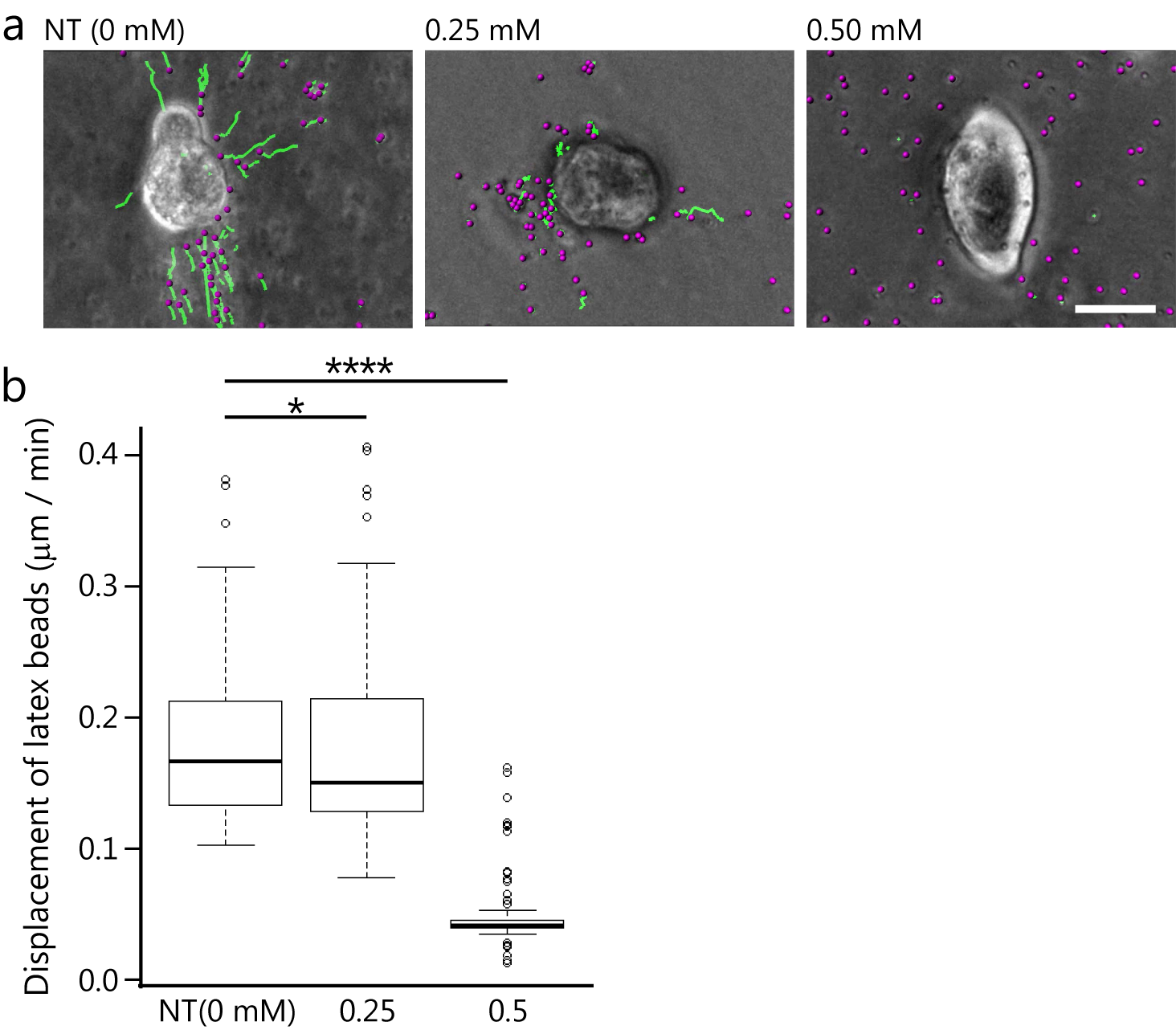


**Supplementary Figure 1**

The matrigel surface is unchanged with increasing concentration of GP during the 3D morphogenesis on GP-Matrigels

(a) Trajectories of latex beads embedded in the GP-matrigel. The beads are shown as magenta spheres, and their trajectories are shown as green lines. The green lines represent the displacement for 1.5 h. For this analysis, more than 30 latex beads were randomly chosen from each condition. Scale bar = 50 μm. (b) The box plot shows the displacement of embedded latex beads. Error bars represent s.e.m. *, p < 0.05, ****, p < 0.0001.


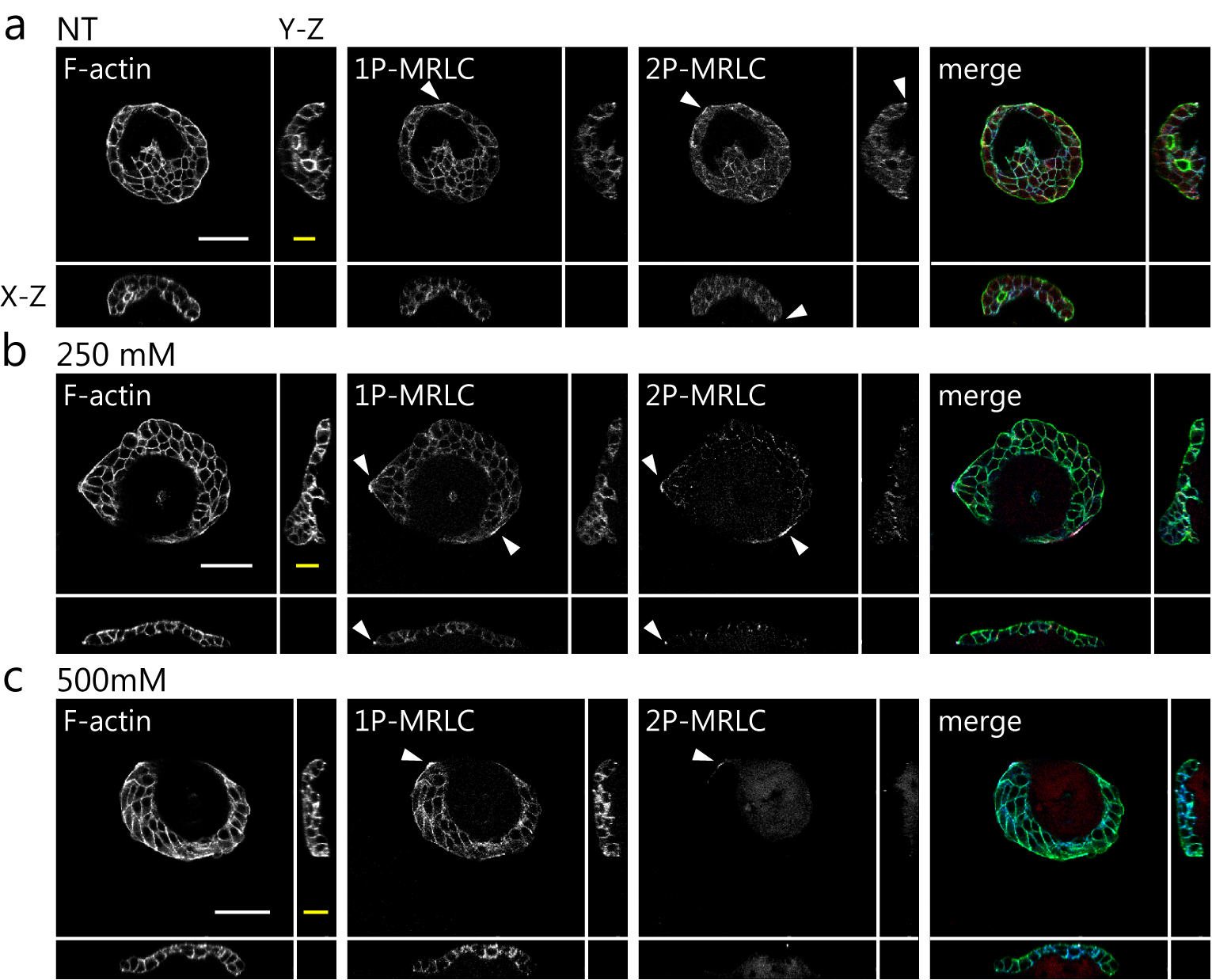


**Supplementary Figure 2**

Localization of phosphorylated-MRLC in the cells cultured on GP-Matrigel.

F-actin, 1P-MRLC, and 2P-MRLC staining on the matrigel surface. MDCK cells cultured on (a) NT-GP-Matrigel (control), (b) 0.25 mM-GP-Matrigel, and (c) 0.50 mM-GP-Matrigel. Cross-sectional views, X-Z plane and Y-Z plane, are shown together. White arrowheads in the images represent 1P-MRLC and 2P-MRLC localization. Three colonies were randomly chosen in each experiment (N = 3). Scale bar (white) = 50 µm and scale bar (yellow) = 20 µm.


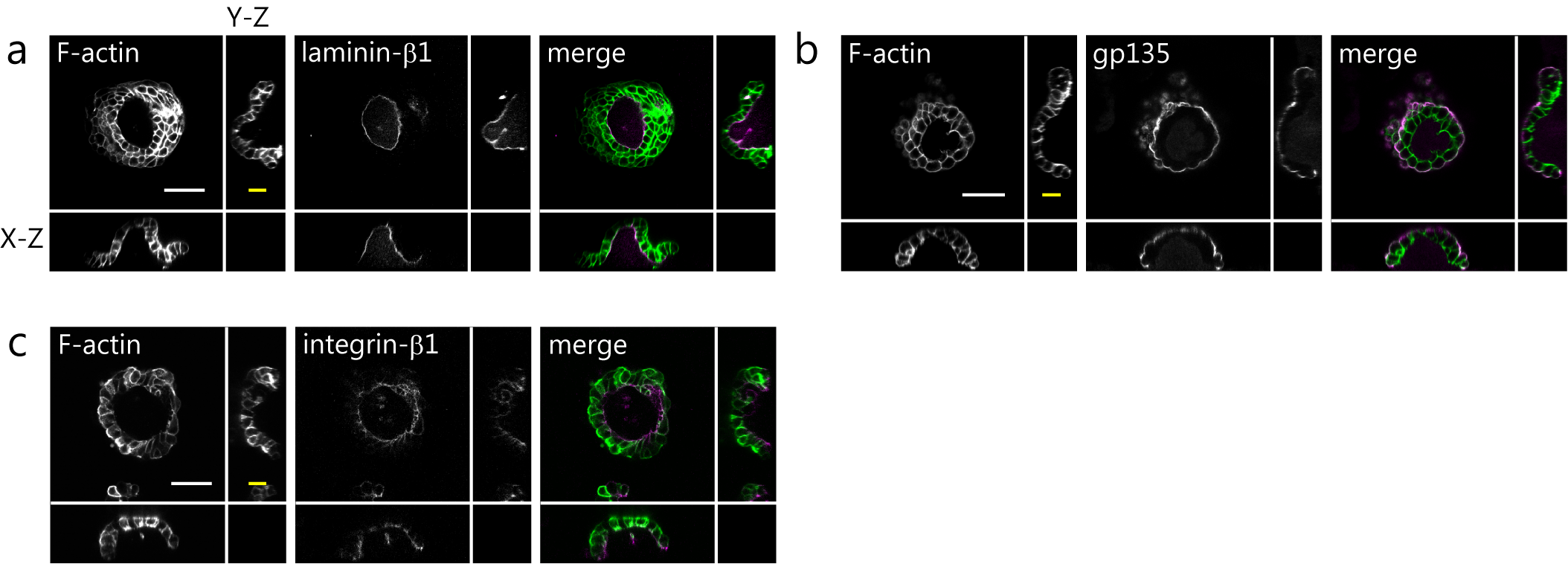


**Supplementary Figure 3**

Epithelial cell apico-basal polarity is involved in the formation of the tulip hat-like structure. Immunofluorescence images of (a) laminin-β1, (b) gp135, and (c) integrin-β1 in MDCK cells cultured on matrigel. Green: F-actin, magenta: (a) laminin-β1, (b) gp135, and (c) integrin-β1, respectively. Scale bar (white) = 50 μm and scale bar (yellow) = 20 µm.


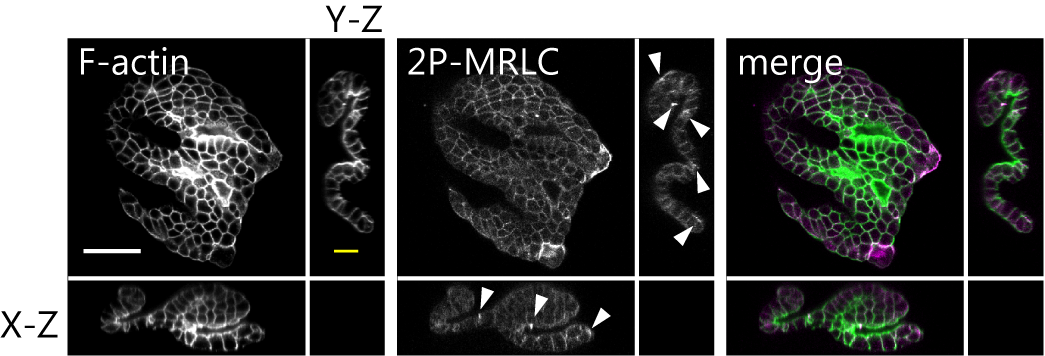


**Supplementary Figure 4**

3D villi-like structure was observed when the flattened epithelial sheets were cultured on the matrigel for 48 h. Green: F-actin, Magenta: 2P-MRLC. White arrowheads in the figure represent 2P-MRLC localization. Scale bar (white) = 50 μm and scale bar (yellow) = 20 μm.

**
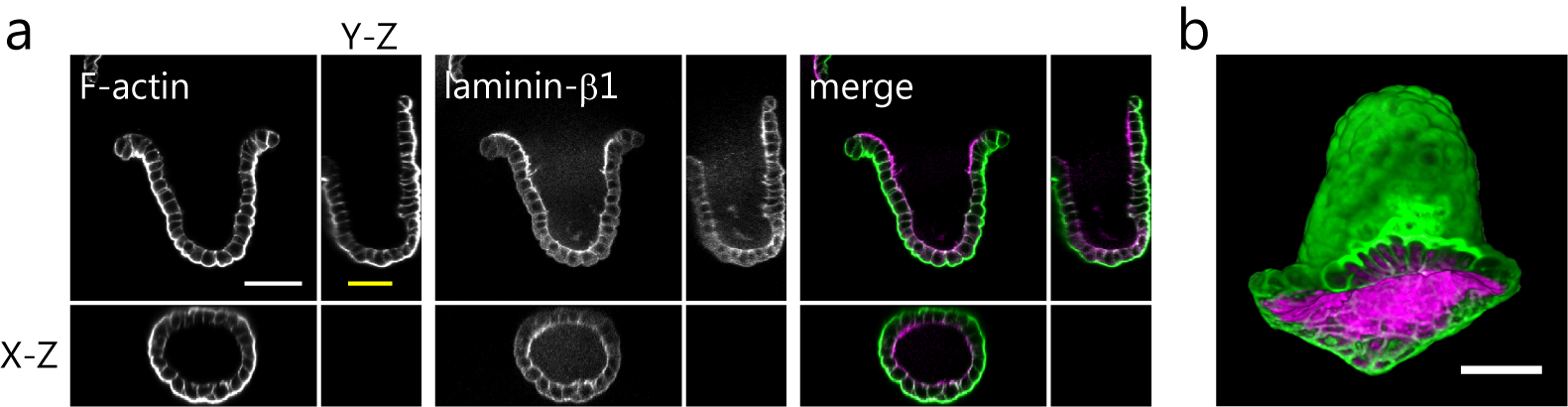
**

**Supplementary Figure 5**

The 3D tulip hat-like morphology was maintained for 2weeks.

(a) Immunofluorescence images of the tulip hat-like morphology of cells cultured on NT-Matrigel. Green: F-actin, Magenta: laminin-β1. Cross-sectional views are shown together. Scale bar = 50 µm. (b) 3D reconstruction image of (a). The image of the 3D tulip hat-like morphology was reconstructed using the results presented in (a). Scale bar (white) = 50 µm and scale bar (yellow) = 20 μm.

Supplemental movies

**Movie S1**

Time-lapse movie of a stainless ball dropped into the matrigel. The numbers in the movie represent GP concentrations. In the movie, 1 s = 300 min in real time.

**Movie S2**

Time-lapse movie of the latex bead tracking analysis, related to Figure 3a, NT

MDCK cells were cultured on matrigel embedded with latex beads. Magenta spheres indicate the position of the latex beads and the green bar shows the beads’ trajectories. In the movie, 1s = 75 min in real time. Bar = 50 μm.

**Movie S3**

Time-lapse movie of the latex bead tracking analysis, related to Figure 3a, Y-27632

The cells were treated with Y-27632 (10 μM). In the movie, 1s = 75 min in real time. Bar = 50 μm.

**Movie S4**

Time-lapse movie of the latex bead tracking analysis, related to Supplementary Figure 1a, NT-GP-Matrigel

MDCK cells were cultured on NT-GP-matrigel embedded with latex beads. Magenta spheres indicate the position of the latex beads, and the green bars show the beads’ trajectories. In the movie, 1s = 75 min in real time. Bar = 50 μm.

**Movie S5**

Time-lapse movie of the latex bead tracking analysis, related to Supplementary Figure 1a, 0.25-GP-Matrigel

MDCK cells were cultured on 0.25-GP-matrigel embedded with latex beads. In the movie, 1s = 75 min in real time. Bar = 50 μm.

**Movie S6**

Time-lapse movie of the latex bead tracking analysis, related to Supplementary Figure 1a, 0.50-GP-Matrigel

MDCK cells were cultured on 0.50-GP-matrigel embedded with latex beads. In the movie, 1s = 75 min in real time. Bar = 50 μm.
